# Low-Level Viremia among Adults Living with HIV on Dolutegravir-Based First-Line Antiretroviral Therapy Is a Predictor of Virological Failure in Botswana

**DOI:** 10.3390/v16050720

**Published:** 2024-05-01

**Authors:** Ontlametse T. Bareng, Sikhulile Moyo, Mbatshi Mudanga, Kagiso Sebina, Catherine K. Koofhethile, Wonderful T. Choga, Natasha O. Moraka, Dorcas Maruapula, Irene Gobe, Modisa S. Motswaledi, Rosemary Musonda, Bornapate Nkomo, Dinah Ramaabya, Tony Chebani, Penny Makuruetsa, Joseph Makhema, Roger Shapiro, Shahin Lockman, Simani Gaseitsiwe

**Affiliations:** 1 Botswana Harvard Health Partnership, Gaborone 0000, Botswanakhei79@gmail.com (C.K.K.); natasha.o.moraka@gmail.com (N.O.M.); dmaruapula@gmail.com (D.M.);; 2 Department of Medical Sciences, Faculty of Allied Health Professions, University of Botswana, Gaborone 0022, Botswanamotswaledims@ub.ac.bw (M.S.M.); 3 Department of Immunology and Infectious Diseases, Harvard T.H. Chan School of Public Health, Boston, MA 02115, USA; 4 Department of Pathology, Division of Medical Virology, Faculty of Medicine and Health Sciences, Stellenbosch University, Cape Town 7935, South Africa; 5 School of Health Systems and Public Health, Faculty of Health Sciences, University of Pretoria, Pretoria 0028, South Africa; 6 Department of Strategic Information, Botswana-University of Maryland School of Medicine Health Initiative, Gaborone 0022, Botswana; 7 Botswana Ministry of Health, Gaborone 0038, Botswanatchebani@gmail.com (T.C.);; 8 Division of Infectious Diseases, Brigham & Women’s Hospital, Boston, MA 02115, USA

**Keywords:** people living with HIV, dolutegravir-based first line antiretroviral therapy, low level viremia, Botswana

## Abstract

We evaluated subsequent virologic outcomes in individuals experiencing low-level virem ia (LLV) on dolutegravir (DTG)-based first-line antiretroviral therapy (ART) in Botswana. We used a national dataset from 50,742 adults who initiated on DTG-based first-line ART from June 2016–December 2022. Individuals with at least two viral load (VL) measurements post three months on DTG-based first-line ART were evaluated for first and subsequent episodes of LLV (VL:51–999 copies/mL). LLV was sub-categorized as low-LLV (51–200 copies/mL), medium-LLV (201–400 copies/mL) and high-LLV (401–999 copies/mL). The study outcome was virologic failure (VF) (VL ≥ 1000 copies/mL): virologic non-suppression defined as single-VF and confirmed-VF defined as two-consecutive VF measurements after an initial VL < 1000 copies/mL. Cox regression analysis identified predictive factors of subsequent VF. The prevalence of LLV was only statistically different at timepoints >6–12 (2.8%) and >12–24 (3.9%) (*p*-value < 0.01). LLV was strongly associated with both virologic non-suppression (adjusted hazards ratio [aHR] = 2.6; 95% CI: 2.2–3.3, *p*-value ≤ 0.001) and confirmed VF (aHR = 2.5; 95% CI: 2.4–2.7, *p*-value ≤ 0.001) compared to initially virally suppressed PLWH. High-LLV (HR = 3.3; 95% CI: 2.9–3.6) and persistent-LLV (HR = 6.6; 95% CI: 4.9–8.9) were associated with an increased hazard for virologic non-suppression than low-LLV and a single-LLV episode, respectively. In a national cohort of PLWH on DTG-based first-line ART, LLV > 400 copies/mL and persistent-LLV had a stronger association with VF. Frequent VL testing and adherence support are warranted for individuals with VL > 50 copies/mL.

## 1. Introduction

Dolutegravir (DTG), a second-generation *integrase* strand transfer inhibitor (INSTI), is part of the preferred regimen for first-line HIV treatment because of its efficacy, tolerability, limited drug–drug interactions, and high barrier to resistance [1,2]. HIV treatment guidelines aim to maximally suppress the VL to undetectable levels [3], which is associated with a positive response to ART compared to detectable viral loads [4]. High viral suppression rates of >90% within 48 weeks of starting DTG-based ART have been reported [5,6,7]. Nevertheless, a subset of people living with HIV (PLWH) experience low-level viremia (LLV) on DTG-based ART, defined as a detectable viral load (VL) between 50–1000 copies/mL [8]. The mechanisms of LLV are not well understood, and may be due to ongoing viral replication in reservoirs due to suboptimal drug penetration, and/or viral production by the activation of HIV-infected cells [9,10], with an uncertain impact of LLV on infection of new cells [9]. Low-level HIV replication during ART may lead to the accumulation of drug-resistance mutations [11,12] and immunologic decline [13], which may be detrimental to future ARV options [14,15]. This highlights the necessity to evaluate the impact of LLV among individuals on DTG-based first-line ART.

Previous work from a limited cohort across Botswana found an association of LLV with subsequent virologic failure [16], but this was during the era of non-nucleoside reverse transcriptase inhibitor (NNRTI)-based first-line ART. We, therefore, aimed to determine the trends of LLV over 5 years among PLWH on DTG-based first-line ART in Botswana and to evaluate LLV and other predictive factors of virologic failure (VF). These analyses may provide insights into treatment outcomes after LLV among DTG-experienced populations and the management of LLV in the era of DTG-based first-line ART.

## 2. Materials and Methods

### 2.1. Study Population

This was a retrospective longitudinal analysis of National ART Program data from all PLWH (aged 18 years and above) who were initiated on tenofovir disoproxil fumarate/emtricitabine//DTG (truvada/DTG) or tenofovir disoproxil fumarate/lamivudine/DTG (TLD) from June 2016 to December 2022 in Botswana. Clinical information from all PLWH in Botswana was captured in the electronic Integrated Patient Management System (IPMS) and/or Patient Information Management System (PIMS), which are used in routine clinical care and operational monitoring and assessment of HIV treatment program.

### 2.2. Viral Load Monitoring in Botswana

According to Botswana HIV treatment guidelines, viral load (VL) testing is performed firstly at three months post ART initiation and every six months thereafter. Individuals who are not suppressed in VL measurement at six months are considered as virological failing after a confirmatory VL is completed and VL testing will be repeated at 4–6 weeks, where those who have 1 Log10 drop will continue with frequent VL measurements at 2 week intervals until viral suppression is achieved. Individuals with no drop in VL will be initiated on directedly observed therapy (DOT) and VL testing will be performed after 30 days on DOT and if suppression is not achieved, HIV drug resistance testing will be conducted. PLWH who are virally suppressed will then have their VLs tested at six-month intervals [17].

### 2.3. Selection of Study Participants

The main study inclusion criteria were starting DTG-based first-line ART in the Botswana National Program from June 2016 to December 2022 in Botswana, being on ART for at least three months and having at least one VL measurement taken after at least 12 weeks following ART initiation. In instances in which a PLWH had a missing ART initiation date and/or missing baseline CD4 count test date, the first VL test date was adapted as the initiation date and then this VL measurement was not included in the proportions of VL groups.

All PLWH who had at least 2 VL measurements were included in the longitudinal analysis. Individuals on ART for at least three months and with first and subsequent LLV after initiation on DTG were followed up until the primary outcome was reached.

### 2.4. Definitions of Exposures and Outcomes

HIV-1 RNA VL measurements were quantified at different laboratories in Botswana using several assays with different lower limits of detection. We defined viral load endpoints/outcomes as VF (VL ≥ 1000 copies/mL), LLV (VL 51–999 copies per mL), or viral suppression (VL ≤ 50 copies/mL) on measurements taken at least three months post ART initiation. Instances of LLV were additionally grouped in ranges of 51–199 copies/mL (low-LLV), 200–400 copies/mL (medium-LLV), and 401–999 copies/mL (high-LLV). PLWH with LLV were further classified into LLV episodes as having a single-LLV (single-LLV episode), confirmed-LLV (two consecutive LLV measurements) within 12 ± 2 months and persistent-LLV (at least three consecutive LLV measurements) 18 ± 2 months. VF was also classified into virologic non-suppression (single instance of VL ≥ 1000 copies/mL) and confirmed VF (two consecutive VL ≥ 1000 copies/mL) within 12 ± 2 months among individuals with VL < 1000 copies/mL. Approximately 140,000 VL measurements reported as 0 or 400 copies/mL were adapted as VL ≤ 50 copies/mL as the prior and post VL measurements were VL ≤ 50 copies/mL within 12 months.

PLWH who experienced VF at their initial VL measurement were not included in this analysis for predictive factors of VF. Individuals with multiple VLs < 1000 copies/mL were assigned to the VL group depending on their highest VL measurement (viral suppression, low-LLV, medium-LLV and high-LLV) before VF was achieved from the time since DTG-based first-line ART initiation within individuals.

### 2.5. Statistical Analysis

We firstly analyzed the prevalence of virologic response categories (e.g., viral suppression [VL ≤ 50 copies/mL], LLV and VF among available VL measurements within each stratum of time since DTG-based first-line ART initiation within individuals. Time since DTG-based first-line ART initiation within an individual was classified into 7 categories: 3–6 months; >6–12 months; >12–24 months; >24–36 months; >36–48 months; >48–60 months; and >60 months. Individuals with multiple VL measurements were classified in the range of the highest VL group result when falling in the same duration strata for the calculation of VL group prevalence.

We then analyzed the association of predictors of VF using univariate and multivariate Cox proportional hazard regression models. The potential predictors of confirmed virologic failure and non-virologic suppression that we included were LLV (classified into LLV ranges and episodes), sex, age, time since DTG-based first-line ART initiation within individuals, DTG-based regimens, baseline CD4 count and Botswana citizenship status. The results were reported as hazard ratios indicating the risk of VF for each variable compared to their reference groups. When assessing the association of LLV with VF, each range of LLV and LLV episodes was compared with virological suppression of VL ≤ 50 copies/mL. In our analysis, PLWH who experienced VF were right censored while those not experiencing VF were right censored after their last available VL. The proportional hazard assumption was checked graphically and by tests based on Schoenfeld residuals. Kaplan–Meier survival curves were used to monitor time to VF among the different variables, which were also displayed with extended Kaplan–Meier estimators, allowing for changing compositions of the exposure groups over time. The log-rank test was used to assess the equality of the survival function of each variable toward VF. *p*-values below 0.05 were considered statistically significant. All of the analyses were conducted using STATA version 15.

## 3. Results

**Figure 1:**
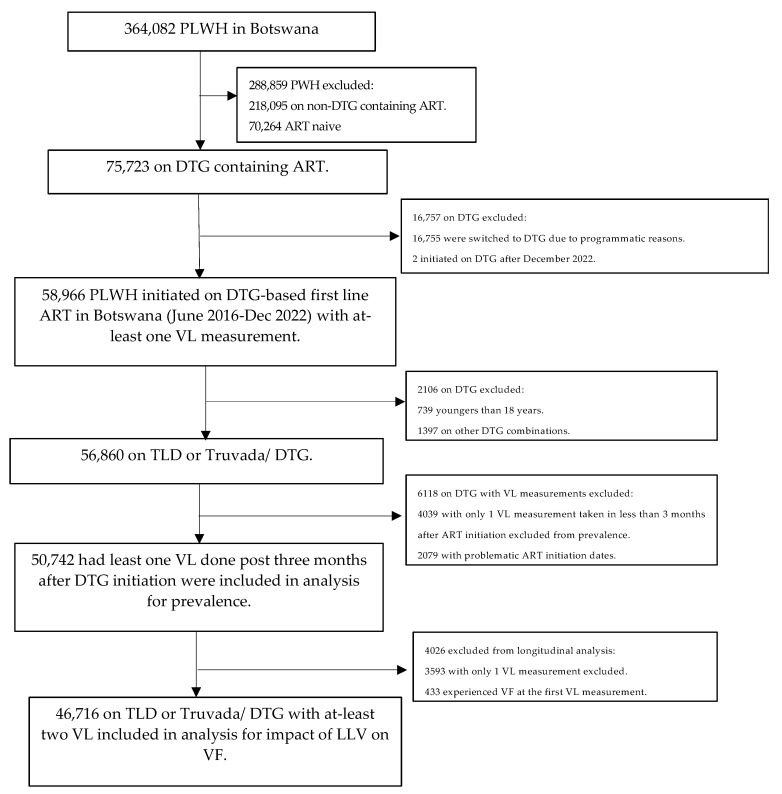
Schema for selection of study participants. ART-antiretroviral therapy, DTG—dolutegravir, LLV—low level viremia, PLWH—people living with HIV, TLD—tenofovir-lamivudine-dolutegravir, VL—viral load.

Among 75,723 individuals who were initiated on DTG-based regimens in Botswana between June 2016 and December 2022, 58,966 were initiated on DTG-based first-line ART for at-least 3 months on ART and all of them had at least one viral load measurement. Among 58,966, we excluded participants < 18 years (739) and non-TLD/non-Truvada DTG combinations (1397). A further 6118 were excluded as 4039 had their initial VL test prior to three months on DTG and 2079 had inconsistent initiation dates resulting in an analysis set of 50,742 PLWH (see Figure 1). All 50,742 PLWH were utilized for the prevalence of VL groups while individuals (46,716) with at least two viral load measurements were utilized in the analysis for the predictive factors of VF.

### 3.1. Demographic Characteristics

Among the 50,742 individuals included, 30,153 (59.4%) were females and the median age was 35.5 (Q1, Q3: 29.0, 43.2) years. The median time since DTG-based first-line ART initiation within individuals was 4.3 (Q1, Q3: 2.5, 5.4) years. The median time from HIV-positive results to ART initiation (days) among PLWH on DTG was 52 (2, 1481) days (Table 1).

### 3.2. Trend of VL Groups across the Years

**Figure 2:**
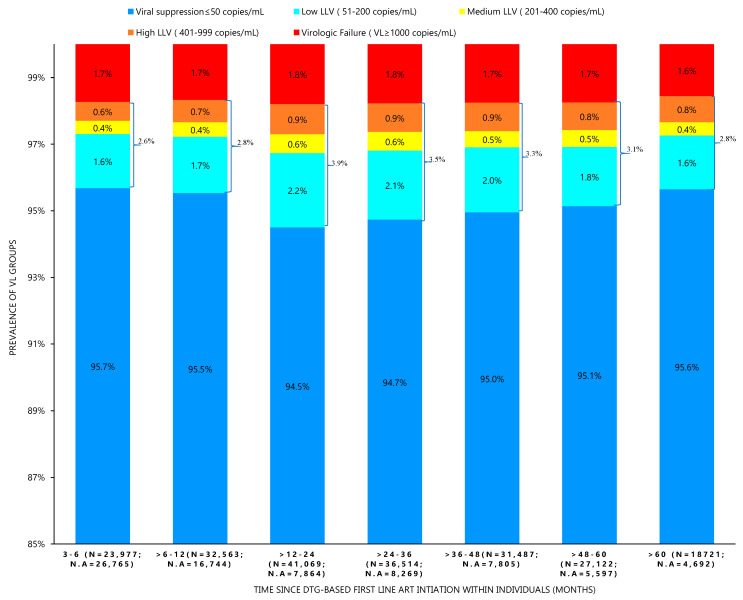
Prevalence of viral load groups by time since DTG-based first-line ART initiation. DTG—dolutegravir, LLV—low level viremia, n—PLWH with VL measurement at the timepoint, n.a—PLWH with no VL at the timepoint, VL—viral load.

Among 50,742 PLWH who were used to determine the prevalence of VL groups, 53.0% (26,765/50,742), 34.0% (16,744/49,307), 16.1% (7864/48,933), 18.5% (8268/44,782), 24.8% (7805/39,292), 17.1% (5597/32,719 and 20.0% (4692/23,413) at timepoints 3–6, >6–12, >12–24, >24–36, >36–48, >48–60 and >60 months, respectively, had no VL measurements available. Amongst 23,977 PLWH who had VL tested within 3–6 months after initiating DTG-based regimens 22,915 (95.7%) were suppressed at VL ≤ 50 copies/mL, 622 (2.6%) had LLV, while 414 (1.7%) had VL ≥ 1000 copies/mL. By time since DTG-based first-line ART initiation within individuals, the viral suppression was 95.5%, 94.5%, 94.7%, 95.0%, 95.1% and 94.5% at timepoints >6–12, >12–24, >24−36, >36–48, >48–60 and >60 months, respectively. The virologic failure rates were 1.7% at months 3–6 and >6–12; 1.8% at months >12–24 and >24–36; and 1.7% at months >36–48 and >48–60, while 1.6% was reported at the >60 months timepoint. The overall prevalence of LLV as a whole group by time after ART start was 2.6%, 2.8%, 3.9%, 3.5%, 3.3%, 3.1% and 2.8% at 3–6, >6–12, >12–24, >24–36, >36–48, >48–60 and >60 months of the follow-up period, respectively (Figure 2). The prevalence of LLV was only statistically different at timepoints >6–12 (2.8%) and >12–24 (3.9%) (*p*-value < 0.01), showing a cross-sectional comparison in these two timepoints. The median time between consecutive VL measurements was 186 (Q1, Q3: 168, 253) days among individuals with at least two VL measurements.

### 3.3. Factors Associated with Virologic Non-Suppression and Confirmed Virologic Failure

**Figure 3:**
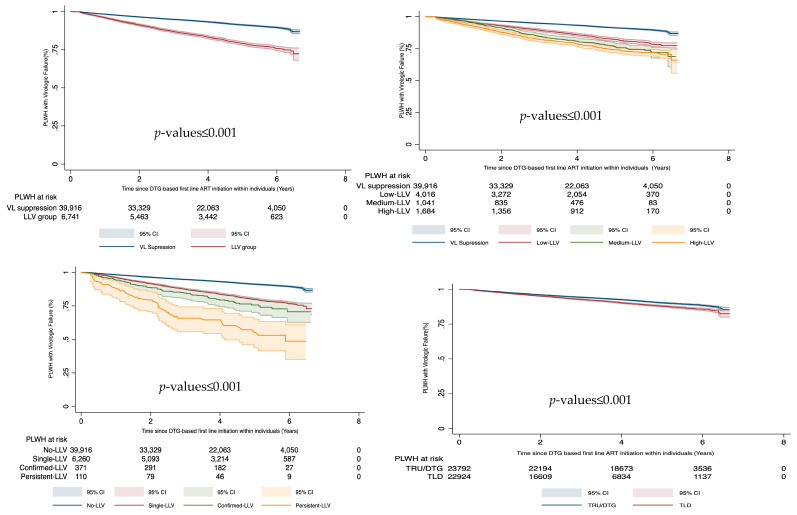
Treatment outcomes among PLWH on DTG based first-line ART. DTG—dolutegravir, LLV—low level viremia, VF—Virologic failure, VL—viral load.

Among 46,716 PLWH on DTG-based first-line ART who had at least two VL measurements taken at least three months after starting ART, 917 (2.0%) experienced virologic non-suppression (a single VL ≥ 1000 copies/mL). Individuals who experienced any LLV were 2.5 (95% CI: 2.4–2.7) times more likely to progress to virologic non-suppression compared to individuals who maintained VL suppression before VF was reached. The hazard ratio of experiencing virologic non-suppression increased with the LLV ranges; individuals with high-LLV had 3.3 (95% CI: 2.9–3.6), medium-LLV with 2.9 (95% CI: 2.5–3.3) and low-LLV had 2.7 (95% CI: 2.3–3.3) hazard ratios compared to individuals with VL below ≤ 50 copies/mL. Individuals with single-LLV, confirmed-LLV and persistent-LLV had statistically increased hazard ratios of 2.2 (95% CI: 2.0–2.3), 3.3 (95% CI: 2.5–3.3) and 6.6 (95% CI: 4.9–8.9), respectively, to present virologic non-suppression compared to PLWH who had not experienced LLV. Experiencing virologic non-suppression was associated with being male (aHR: 1.3 (95% CI: 1.2–1.5)), age younger than 45 years old and having a baseline CD4 count in the lower quartile (aHR: 1.7 (95% CI: 1.5–2.0)). The use of TLD was independently associated with virologic non-suppression, but when adjusting for other variables, the association was not statistically significant. A one-year increase in time since initiating DTG-based first-line ART within individuals was associated with a 70% reduction in the hazard ratios of virologic non-suppression (aHR: 0.4 (95% CI: 0.3–0.4) (Table 2, Figure 3).

Confirmed VF occurred in 149 (0.3%) PLWH. LLV was also associated with confirmed VF (aHR: 3.7 (3.0–4.5)) when adjusting for age, gender, and time since DTG-based first-line ART initiation within individuals and baseline CD4 count, while being male was not associated with an increased hazard ratios of presenting confirmed VF (Table 3). Low survival probability was reported in low level viremia compared to virally suppressed (Supplementary Table S1), high-LLV against low-LLV (Supplementary Table S1) and persistent-LLV compared to a single instance of LLV (Supplementary Table S1).

## 4. Discussion

We analyzed the trends of LLV over a period of 5 years in Botswana to determine whether LLV after starting DTG-based ART is associated with subsequent virologic failure. National program data from adults living with HIV who initiated DTG-based first-line ART revealed high suppression rates of greater than 94.0%. Despite these high suppression rates, we observed that the occurrence of LLV after starting DTG-based first-line ART was strongly associated with both subsequent virologic non-suppression and confirmed virologic failure among individuals on DTG-based first-line ART. High VL suppression is expected during this evaluation period since Botswana was among the first countries to adopt the use of DTG-based first-line ART in June 2016, which was accompanied by the implementation of a treat-all strategy regardless of CD4+ T cell count [18]. In 2022, Botswana became one of the first three countries to exceed the 95:95:95 UNAIDS goals [19]. Our findings also emphasize that Botswana has reached virologic suppression targets even when the virologic suppression threshold is lowered to 50 copies/mL. Despite this, approximately 4.0% of PLWH are not suppressed on DTG-based first-line ART, many of whom experienced prior LLV.

The lowest prevalence of LLV reported in the study was 2.6% while the highest was 3.9%, at >3–6 and >12–24 months’ time since ART initiation within individuals. This prevalence was lower when compared to another real observational study conducted in India (LLV was 17.9%, 23.3% and 26.9% at 6, 12, 18 and 24 months) [20]. A higher LLV prevalence was also reported in a study performed in Nigeria: 1 (15.8%), 2 (15.6%), 3 (14.8%), 4 (13.7%), 5 (13.3%) and 6 (11.3%) annually since ART initiation [21]. The LLV prevalence reported in our study was in the same range as rates reported in Uganda, although LLV was reported from 2016–2020 (calendar years) instead of annually [22]. Our study only focused on adults living with HIV who were on DTG-based first-line ART compared to other studies that included all PLWH on DTG including those who are on second-line therapy [20,21,22], which could have led to a lower prevalence of LLV in our study.

We report that individuals with LLV are at risk of presenting with virologic non-suppression and confirmed virologic failure. The findings indicate that any LLV category or episode such as low-LLV and a single instance of LLV are independent predictors of future VF. This highlights the need for interventions to eliminate LLV, which may have negative long-term virologic outcomes. Unstable VL suppression has been linked to HIV transmission events, with a few cases reported at detectable VL > 1000 copies/mL [23]. This shows that the HIV treatment guidelines that initiated adherence support care at VL > 200 copies/mL are most likely to miss PLWH with VL: 50–200 copies/mL who are at risk of presenting virologic non-suppression and confirmed virologic failure. Therefore, extending intensified adherence support to PLWH with detectable VL > 50 copies/mL to ensure that viral suppression is achieved is crucial for the prevention and control of new HIV infections. The frequent viral load monitoring of PLWH with LLV can provide a further understanding of HIV transmission risk at LLV.

While low-LLV was associated with VF, there was an increased risk of presenting VF among individuals with high-LLV and persistent-LLV. Our results support the previous studies [24,25]. Some of these studies used a lower VF threshold between 400–500 copies/mL [26]. Similarly, LLV has been associated with the presence of HIV drug resistance even in a single instance of LLV [15,16]. These results highlight the need for HIV drug resistance testing at persistent LLV for the selection of appropriate ART options that will further avoid accumulations of more drug resistance mutations. Persistent-LLV has been associated with immune activation and inflammation resulting in non-AIDS defining events [27], viral reservoir size [28] and clonal expansion [29]. Studies on mechanisms of LLV in association with ongoing viral replication in reservoirs due to suboptimal drug penetration, and/or viral production by the activation of HIV-infected cells are warranted to further understand the virologic and immunological outcomes of LLV.

Our study has several limitations. We evaluated the viral suppression rates starting at 3 months on DTG-based first-line ART, which could have underestimated VL groups as some PLWH might have still been on their way to VL suppression. However, there are clinical trials that have reported high suppression rates among PLWH on DTG within a short period of less than 3 months [30,31]. The use of real-world data can lead to selection bias when selecting the PLWH who meet study inclusion criteria, leading to some PLWH being excluded from the study and missing data. The median proportion of PLWH with missing VL measurements was 20.0% across the seven timepoints, which could have led to an underestimation of the prevalence of LLV and VF. We did not perform a sensitivity analysis to determine the impact of missingness, as PLWH were removed based on the initiation date on DTG and availability of VL measurement; however, we retained relatively large numbers for the analysis. These missing data could have been due to missed visits, missed tests or general loss to follow-up. The VL measurements were performed in different laboratory set-ups using assays with different limits of detection and quantification. Approximately 140,000 VL measurements that were reported as 0 or 400 copies/mL were adapted as VL ≤ 50 copies/mL in our analysis; this could have led to a reduced prevalence of LLV and affected the comparison of LLV at lower LLV ranges. However, this was based on the VL ≤ 50 copies/mL prior and post these VL measurements. Lack of adjusting for adherence in the predictive factors associated with VF could have led to an increased risk of VF among individuals with LLV. Unavailability of HIV drug resistance data of individuals with LLV hindered the ability to determine if a lack of suppression resulted from the presence of HIV drug resistance mutations or other factors. Our study utilized a large sample size with approximately 310,000 viral load records from 50,742 PLWH in Botswana, providing a good representative sample of PLWH on DTG based first line ART in Botswana and providing insights into virologic outcomes among individuals with LLV on DTG-based first-line ART.

## 5. Conclusions

In a large-scale cohort of PLWH on DTG-based first-line ART in Botswana, the prevalence of LLV ranged from 2.6% to 3.9%. Our results show that LLV is strongly associated with future VF, particularly high-LLV and persistent-LLV. These findings show that any VL > 50 copies/mL may lead to poorer treatment outcomes, therefore highlighting the importance of using VL ≥ 50 copies/mL as an indicator for intensified adherence care support and frequent VL monitoring.

## Figures and Tables

**Table 1:** Characteristics of participants included in the study.

Variables	Total PLWH on DTG n = 50,742 (%)
Sex	
Male	20,589 (40.6)
Female	30,153 (59.4)
Median age (years) (Q1, Q3) at ART initiation	35.5 (29.0, 43.2)
<25	6491 (12.8)
25–34.9	17,773 (35.0)
35–44.9	15,689 (30.9)
45–54.9	6950 (13.7)
55–64.9	2921 (5.8)
≥65	918 (1.8)
Year of ART initiation	
2016	10,065 (19.8)
2017	13,348 (26.3)
2018	9306 (18.3)
2019	6573 (13.0)
2020	5491 (10.8)
2021	4150 (8.2)
2022	1809 (3.6)
Median time since DTG-based ART initiation (years) (Q1, Q3)	4.3 (2.5, 5.4)
Median number of VL measurements during follow-up (at-least 3 months after ART start) (Q1, Q3)	4 (2, 8)
Median duration of ART at first VL measurement (Q1, Q3) (months)	7.3 (4.5, 12.8)
Botswana Citizen	
Yes	48,496 (95.6)
No	2181 (4.3)
Spouse to citizen	65 (0.1)
Median baseline (pre-ART) CD4 count (Q1, Q3) (n = 23,913)	343 (200, 511)
Median duration from positive HIV test to ART initiation (Q1, Q3) (days)	56 (2, 1481)

ART—antiretroviral therapy, DTG—dolutegravir, LLV—low level viremia, PLWH—people with HIV, TLD—tenofovir lamivudine dolutegravir, VL—viral load, Q1—First quartile, Q3—Third quartile.

**Table 2:** Factors associated with virologic non-suppression among PLWH on DTG-based first-line ART in Botswana.

Risk Factor	Virologic Failure Group (*n* = 923)	Non-Virologic Failure Group (*n* = 45,793)	Univariate HR (95% CI)	*p*-Values	Multivariate HR (95% CI)	*p*-Values
Viral load group						<0.001
Suppressed (n = 39,975)	696 (1.7)	39,297 (98.3)	1 (ref)		1 (ref)	
low level viremia (n = 6741)	227 (3.4)	6514 (96.6)	2.5 (2.4–2.7)	<0.001	2.4 (2.2–2.7)	
VL ranges					*	
VL ≤ 50 copies/mL (n = 39,975)	699 (1.7)	39,297 (98.3)	1 (ref)			
Low LLV (51–200) (n = 4016)	117 (2.9)	3899 (97.0)	2.1 (2.0–2.3)	<0.001		
Medium LLV (201–400) (n = 1041)	43 (4.1)	998 (95.9)	2.9 (2.5–3.3)	<0.001		
High LLV (401–999) (n = 1684)	67 (4.0)	1617 (96.0)	3.3 (2.9–3.6)	<0.001		
Low-level viremia episodes					*	
No LLV (n = 39,975)	696 (1.7)	39,297 (98.3)	1 (ref)			
Single LLV (n = 6260)	204 (3.3)	6056 (96.7)	2.4 (2.3–2.6)	<0.001		
Confirmed (n = 371)	15 (4.0)	356 (96.0)	3.1 (2.5–3.9)	<0.001		
Persistent (n = 110)	8 (7.3)	102 (92.7)	6.6 (4.9–8.9)	<0.001		
Sex						
Female (n = 28,076)	524 (1.9)	27,552 (98.1)	1 (ref)	<0.001	1 (ref)	<0.001
Male (n = 18,640)	399 (2.1)	18,241 (97.9)	1.2 (1.1–1.3)		1.4 (1.3–1.5)	
Age						
<25 (n = 5923)	214 (3.6)	5709 (96.4)	2.6 (2.2–3.0)	<0.001	3.2 (2.6–4.0)	<0.001
25–34.9 (n = 16,387)	365 (2.2)	16,022 (97.8)	1.7 (1.5–2.0)	<0.001	2.0 (1.6–2.4)	<0.001
35–44.9 (n = 14,448)	229 (1.6)	14,219 (98.4)	1.3 (1.2–1.6)	<0.001	1.4 (1.2–1.8)	<0.001
45–54.9 (n = 6373)	79 (1.2)	6294 (98.8)	1.1 (0.9–1.3)	0.44	1.1 (0.8–1.4)	0.54
≥55 (n = 3585)	36 (1.0)	3549 (99.0)	1 (ref)		1 (ref)	
DTG-containing regimen						
Truvada/DTG (n = 23,792)	440 (1.8)	23,352 (98.2)	1 (ref)	<0.001	1 (Ref)	0.10
TLD (n = 22,924)	483 (2.1)	22,441 (97.9)	1.3 (1.2–1.4)		1.1 (1.0–1.2)	
Median time since DTG-based first-line ART initiation (years) (Q1, Q3)	2.5 (1.2–4.0)	2.5 (1.3–4.1)	0.3 (0.3–0.4)	<0.001	0.3 (0.3–0.4)	<0.001
Baseline CD4 counts in quartiles (mm^3^)						
Quartiles 1	187	200	2.0 (1.7–2.2)	<0.001	1.7 (1.5–2.0)	<0.001
Quartile 2	297	350	1.7 (1.5–1.9)	<0.001	1.3 (1.2–1.5)	<0.001
Quartile 3	419	519	1.3 (1.1–1.5)	<0.001	1.2 (1.0–1.4)	0.002
Quartile 4	1020	1183	1 (ref)		1 (ref)	
Botswana citizen					**	
Yes (n = 45,071)	896 (2.0)	44,186 (98.0)	1 (ref)	0.82		
No (n = 1645)	27 (1.6)	1613 (98.4)	1.0 (0.8–1.3)			

DTG—dolutegravir, LLV—low level viremia, n—number of PLWH in each variable, 95% CI—95% confidence intervals, *—excluded due to collinearity. **—Univariate *p*-value ≥ 0.2.

**Table 3:** Factors associated with confirmed VF among PLWH on DTG-based first-line ART in Botswana.

Risk Factor	Virologic Failure Group (*n* = 149)	Non-Virologic Failure Group (*n* = 46,567)	Univariate HR (95% CI)	*p*-Values	Multivariate HR (95% CI)	*p*-Values
Viral load group						
Suppressed (n = 39,975)	103 (0.2)	39,872 (99.8)	1 (ref)		1 (ref)	
low level viremia (n = 6741)	46 (0.7)	6695 (99.3)	3.7 (3.2–4.3)	<0.001	3.7 (3.0–4.5)	<0.001
VL ranges					*	
VL ≤ 50 copies/mL (n = 39,975)	103 (0.2)	39,872 (99.8)	1 (ref)			
Low LLV (51–200) (n = 4016)	18 (0.4)	3998 (99.6)	2.7 (2.3–3.3)	<0.001		
Medium LLV (201–400) (n = 1041)	12 (1.2)	1029 (98.8)	4.6 (3.5–6.0)	<0.001		
High LLV (401–999) (n = 1684)	16 (1.0)	1668 (99.0)	5.5 (4.5–6.8)	<0.001		
Low-level viremia episodes					*	
No LLV (n = 39,975)	103 (0.2)	39,872 (99.8)	1 (ref)			
Single LLV (n = 6260)	39 (0.6)	6221 (99.4)	3.5 (3.0–4.0)	<0.001		
Confirmed (n = 371)	4 (1.1)	367 (98.9)	5.5 (3.6–8.2)	<0.001		
Persistent (n = 110)	3 (2.7)	107 (97.3)	13.4 (8.4–21.4)	<0.001		
Sex						
Female (n = 28,076)	103 (0.4)	27,973 (99.6)	1 (ref)		1 (ref)	
Male (n = 18,640)	46 (0.2)	18,594 (99.8)	0.9 (0.8–1.1)	0.26	1.0 (0.8–1.2)	0.9
Age						
<25 (n = 5923)	40 (0.7)	5883 (99.3)	3.5 (2.4–5.0)	<0.001	3.3 (2.0–5.4)	<0.001
25–34.9 (n = 16,387)	61 (0.4)	16,326 (99.6)	2.0 (1.4–2.8)	<0.001	1.9 (1.2–3.1)	0.007
35–44.9 9 (n = 14,448)	28 (0.2)	14,420 (99.8)	1.6 (1.1–2.3)	0.012	1.5 (0.9–2.5)	0.09
45–54.9 (n = 6373)	15 (0.2)	6358 (99.8)	1.2 (0.8–1.9)	0.15	1.3 (0.8–2.2)	0.33
≥55 (n = 3585)	5 (0.1)	3580 (99.9)	1 (ref)		1 (ref)	
DTG-containing regimen.						0.031
Truvada/DTG (n = 23,792)	59 (0.2)	23,733 (99.8)	1 (ref)		1 (ref)	
TLD (n = 22,924)	90 (0.4)	22,834 (99.6)	1.4 (1.3–1.7)	<0.001	1.2 (1.0–1.5)	
Median time since DTG-based first-line ART initiation (years) (Q1, Q3)	2.2 (0.9–3.8)	2.5 (1.3–4.1)	0.3 (0.3–0.4)	<0.001	0.3 (0.3–0.4)	<0.001
Baseline CD4 counts in quartiles (mm^3^)						
Quartiles 1	166	200	2.9 (2.2–3.9)	<0.001	2.7 (2.0–1.5)	<0.001
Quartile 2	283.5	349	1.8 (1.3–2.5)	<0.001	1.5 (1.1–2.1)	0.02
Quartile 3	448	518	1.7 (1.2–2.3)	0.002	1.7 (1.2–2.3)	0.003
Quartile 4	1040	1182	1 (ref)		1 (ref)	
Botswana citizen					**	
Yes (n = 45,071)	146 (0.3)	44,925 (99.7)	1 (ref)			
No (n = 1645))	3 (0.2)	1642 (99.8)	0.7 (0.4–1.3)	0.27		

DTG—dolutegravir, LLV—low level viremia, n—number of PLWH in each variable, 95% CI—95% confidence intervals, *—excluded due to collinearity. **—Univariate *p*-value ≥ 0.2.

## Data Availability

All relevant data are within the paper, including the figures and tables. No new primary data were collected for this study. The data utilized in this manuscript belong to the Botswana Ministry of Health.

## Supplementary Materials

The following supporting information can be downloaded at: https://www.mdpi.com/article/10.3390/v16050720/s1, Table S1: Low survival probability was reported in low level viremia compared to virally suppressed, high-LLV against low-LLV and persistent-LLV compared to a single instance of LLV.
