# Supplementary material for: Low-Level Viremia among Adults Living with HIV on Dolutegravir-Based First-Line Antiretroviral Therapy Is a Predictor of Virological Failure in Botswana
Source: Viruses. 2024 May 1;16(5):720. doi: 10.3390/v16050720 (PMC11125697; doi:10.3390/v16050720)
Supplement: Supplementary file 1 [file viruses-16-00720-s001.zip › viruses-2946546-supplementary.pdf]

Supplementary Table S1a. Survival Probability by Viral load groups.

Years	Viral Suppression	Whole LLV
0	1.00	1.00
1	0.98	0.97
2	0.96	0.92
3	0.95	0.87
4	0.93	0.84
5	0.91	0.80

Supplementary Table S1b. Survival Probability by low-level viremia (LLV) ranges.

Years	Viral Suppression	Low-LLV	Medium-LLV	High-LLV
0	1.00	1.00	1.00	1.00
1	0.98	0.97	0.97	0.94
2	0.96	0.93	0.92	0.88
3	0.95	0.90	0.86	0.83
4	0.93	0.86	0.82	0.79
5	0.91	0.82	0.77	0.75

Supplementary Table S1c. Survival Probability by the frequency of low-level viremia (LLV).

Years	No-LLV	Single-LLV	Confirmed-LLV	Persistent-LLV
0	1.00	1.00	1.00	1.00
1	0.98	0.96	0.95	0.90
2	0.96	0.92	0.89	0.80
3	0.95	0.88	0.85	0.68
4	0.93	0.84	0.79	0.66
5	0.91	0.80	0.76	0.56
